# Relationship between Serum and Brain Carotenoids, **α**-Tocopherol, and Retinol Concentrations and Cognitive Performance in the Oldest Old from the Georgia Centenarian Study

**DOI:** 10.1155/2013/951786

**Published:** 2013-06-09

**Authors:** Elizabeth J. Johnson, Rohini Vishwanathan, Mary Ann Johnson, Dorothy B. Hausman, Adam Davey, Tammy M. Scott, Robert C. Green, L. Stephen Miller, Marla Gearing, John Woodard, Peter T. Nelson, Hae-Yun Chung, Wolfgang Schalch, Jonas Wittwer, Leonard W. Poon

**Affiliations:** ^1^Jean Mayer US Department of Agriculture Human Nutrition Research Center on Aging at Tufts University, Boston, MA 02111, USA; ^2^University of Georgia-Athens, Athens, GA 30602, USA; ^3^Temple University, Philadelphia, PA 19122, USA; ^4^Harvard University, Boston, MA 02115, USA; ^5^Emory University, Atlanta, GA 30322, USA; ^6^Wayne State University, Detroit, MI 48202, USA; ^7^University of Kentucky, Lexington, KY 40536, USA; ^8^Yonsei University, Seoul 120-749, Republic of Korea; ^9^DSM Nutritional Products, CH-4002 Basel, Switzerland

## Abstract

Oxidative stress is involved in age-related cognitive decline. The dietary antioxidants, carotenoids, tocopherols, and vitamin A may play a role in the prevention or delay in cognitive decline. In this study, sera were obtained from 78 octogenarians and 220 centenarians from the Georgia Centenarian Study. Brain tissues were obtained from 47 centenarian decedents. Samples were analyzed for carotenoids, *α*-tocopherol, and retinol using HPLC. Analyte concentrations were compared with cognitive tests designed to evaluate global cognition, dementia, depression and cognitive domains (memory, processing speed, attention, and executive functioning). Serum lutein, zeaxanthin, and *β*-carotene concentrations were most consistently related to better cognition (*P* < 0.05) in the whole population and in the centenarians. Only serum lutein was significantly related to better cognition in the octogenarians. In brain, lutein and *β*-carotene were related to cognition with lutein being consistently associated with a range of measures. There were fewer significant relationships for *α*-tocopherol and a negative relationship between brain retinol concentrations and delayed recognition. These findings suggest that the status of certain carotenoids in the old may reflect their cognitive function. The protective effect may not be related to an antioxidant effect given that *α*-tocopherol was less related to cognition than these carotenoids.

## 1. Introduction

Cognitive decline in the elderly is a significant public health issue. It has been estimated that the incidence of mild cognitive impairment (MCI) is approximately 19% in those younger than 75 years and 29% in those older than 85 years [1]. Further, 13% of people aged 65 years and older are afflicted with Alzheimer's disease. Studies in centenarians have reported considerable dementia, ranging from 42 to 100% [2, 3]. The number of individuals so affected is likely to increase given that the number of people over 65 years is rising. As with most age-related diseases, the most cost effective way to combat disease is through prevention. One possible strategy is nutrition intervention [4].

Fruit and vegetable intake has been associated with cognitive function [5–7]. For example, in a study of 13,388 women, it was found that total vegetable intake was significantly associated with reduced cognitive decline [8]. The strongest association was with greater intake of green leafy and cruciferous vegetables. Fruits and vegetables are major dietary sources of carotenoids. Carotenoids are a class of naturally occurring pigments that are synthesized by plants and produce the red, orange, and yellow colors of fruits and vegetables. Carotenoids are comprised of two subclasses: xanthophylls (lutein, zeaxanthin, and *β*-cryptoxanthin) and carotenes (*α*-carotene, *β*-carotene, and lycopene). In the Nurses' Health Study, among nonsupplement users, women in the highest quartile of plasma carotenoids had better cognitive performance than those in the lowest quartile [9]. Research has shown that patients with MCI had decreased plasma levels of antioxidants, including carotenoids [10]. Given that dietary carotenoids function as both antioxidants and anti-inflammatory agents and that oxidative stress and inflammation are believed to be involved in the pathogenesis of cognitive decline [11–20], intake of these dietary components may hold promise in cognitive health for the elderly.

The first objective of this study was to evaluate the relationship between serum concentrations of carotenoids and cognitive function in subjects from the Georgian Centenarian Study, a population-based multidisciplinary study of octogenarians and centenarians conducted in Georgia (USA) [21].

Given that an intervention with lutein was reported to improve cognitive function in the elderly [22] and that compared to carotenes, xanthophylls are preferentially taken up into brain tissue [23], a second objective of this research was to evaluate the cross-sectional relationship between brain carotenoids and premortem measures of cognitive function in a subgroup of the centenarian participants. For both serum and brain tissues, *α*-tocopherol was measured for comparison since it crosses the blood brain barrier and has antioxidant properties. Serum and brain retinol concentrations were measured because of the provitamin A activity of certain carotenoids.

This study provides a unique advantage of being able to assess the relationship between serum and brain carotenoids. If indeed serum concentrations of individual carotenoids reflect their levels in the brain and these brain carotenoids are related to better cognitive performance, serum carotenoid measures could be a useful tool for evaluating the benefits of dietary carotenoids to age-related cognitive health.

## 2. Materials and Methods

### 2.1. Study Population

The Georgia Centenarian Study (GCS) [21], a population-based multidisciplinary study conducted in 44 counties in northern Georgia (USA) from 2001 to 2009, was designed to identify and isolate longevity genes, neuropathology, functional capacity, and adaptational characteristics of centenarians [17]. Living status was classified as community dwelling or institutionalized where community dwelling included those living in private residences and institutionalized included individuals living in a skilled nursing facility or personal care home. The study involving serum analyses included 244 centenarians (defined in this study as age 98 yrs and older) and 80 octogenarians. The study involving brain tissue analyses included 47 centenarians who volunteered to donate their brain upon death. Subjects were recruited from the community, personal care homes, and skilled nursing facilities. The sample procedures and data collection methods have been described elsewhere [21].

In the analyses of serum, we excluded 2 octogenarians and 7 centenarians from whom we were unable to obtain sufficient serum for analysis. The final number of subjects with a complete dataset for serum analytes and cognitive function was 220 subjects in the centenarian group and 78 subjects in the octogenarian group.

Brain tissue was obtained from four regions of the brain: right cerebellum, right temporal cortex, and right and left frontal and occipital cortices from the subset of centenarians. Serum and tissues were stored at −80°C until analysis.

### 2.2. Serum and Brain Carotenoids, *α*-Tocopherols, and Retinoids Extraction

Serum was as described previously [24]. The brain extraction procedure was adapted from Park et al. [25] and has been described in detail by our laboratory [26].

### 2.3. HPLC Analysis for Carotenoid, Tocopherols, and Retinol

Serum and brain extracts were analyzed by HPLC (Alliance 2695l Waters, Milford, MA, USA) as previously described [24]. Using this method, *cis *lutein, all-*trans *lutein, *cis* zeaxanthin, all-*trans *zeaxanthin, cryptoxanthin, *α*-carotene, 13-*cis β*-carotene, all-*trans β*-carotene, 9-*cis β*-carotene, *cis *lycopene, and *trans *lycopene were separated and detected at 455 nm. *α*-Tocopherol, and retinol were detected at 292 and 340 nm, respectively. Using this method, the lower limit of detection was 0.2 pmol for carotenoids, 2.7 pmol for *α*-tocopherol and 2.0 pmol for retinol.

The analysis of serum and brain tissues was conducted without knowledge of values for the associated measures of cognition.

### 2.4. Measures of Cognitive Performance

Subjects underwent a battery of cognitive tests designed to evaluate global cognitive function, dementia, and depression as well as several cognitive domains including memory, processing speed, or attention and executive functioning (see Table S1 in the Supplementary Material available online at http://dx.doi.org/10.1155/2013/951786). The Geriatric Depression Scale-short form was administered to screen for depressive symptoms in the subjects [27]. Cognitive measures included the Mini-Mental State Examination (MMSE) [28], Global Deterioration Rating Scale (GDRS) [29], Severe Impairment Battery (SIB) [30], Fuld Object Memory Evaluation (FOME) [31], Wechsler Adult Intelligence Scale-III (WAIS-III) Similarities Subtest [32], Behavioral Dyscontrol Scale [33], and Controlled Oral Word Association Test (COWAT) [34].

For participants in the separate brain donation component, additional cognitive tests were administered every six months until mortality. These tests included the Consortium to Establish a Registry for Alzheimer's Disease (CERAD) neuropsychological battery, which is composed of five subtests derived from previously established cognitive tests (verbal fluency, Boston Naming Test, MMSE, Constructional Praxis, and Word List Memory) [35, 36]. These subtests have been found to be valid and reliable measures of cognition in normal aging and in Alzheimer's disease [37]. Further, no differences were found between participants in the brain donation component of the GCS and the rest of the centenarian participants [38]. All of these tests or versions of them have been used and validated in aging research settings or have demonstrated sensitivity to health variables in epidemiological studies [39–42].

### 2.5. Covariates and Predictors

In the analyses involving serum, covariates and predictors included age (80–89 or ≥98 y), gender, and race (white or African American, by design). The proportion of participants from each age group recruited from skilled nursing facilities was based on estimates of the “institutionalized” population of the study area according to the 2000 US Census figures [21]. Residence status was not considered as a covariate because it was a potential suppressor variable. Fifteen percent of the octogenarians and 62% of the centenarians resided in skilled nursing facilities. The remaining “community dwelling” participants resided in private residences and personal care homes. In the analyses involving brain tissue, which involved centenarian decedents, 31% of the decedents had resided in skilled nursing facilities.

### 2.6. Statistical Analyses

Results are expressed as geometric means ± SDs. Relationships between trans and cis isomers of individual carotenoids and cognition did not differ appreciably. Therefore, the total (*trans* + *cis*) was used in the analysis. Given that the purpose of our analyses was to increase the precision with which an association could be estimated following adjustment for variables associated with our criterion, but not our predictor, we chose to analyze relationships between carotenoids, *α*-tocopherol, and retinol with cognitive measures using partial correlations. Thus, the partial correlation can provide an estimate of substantive interest but has the added advantage in that it does so in a standardized and easily interpretable metric. Statistical significance was set at *P* < 0.05. All statistical analyses were performed using SAS version 9.0 (serum) and SPSS version 19.0 (brain).

#### 2.6.1. Serum Analyses

Data were verified for normality (Shapiro-Wilk test) and, when necessary, were log-transformed for normal distribution before further statistical analysis. Chi-square test and Student's *t*-test were used to compare subject characteristics, serum carotenoid levels, and cognitive values between groups. Pearson's correlations were performed to identify associations between cognitive indices with age, sex, anthropometric variables, and other possible confounders. The associations between cognitive indices and serum carotenoids were determined by calculating partial Pearson's correlation coefficients adjusted for age, sex, body mass index (BMI), smoking, alcohol, diagnosed hypertension, and diabetes. For the centenarians, diagnosis of hypertension and diabetes was drawn from proxy, family, staff, or charts.

#### 2.6.2. Brain Analyses

Data were analyzed for all 47 decedents together and also separately for decedents based on their premortem GDRS scores. The purpose was to determine differences between decedents who had intact cognitive function (GDRS = 1), mild memory loss (GDRS = 2), mild cognitive impairment (GDRS = 3), and dementia (GDRS > 3) before death. One-way ANOVA was used to determine differences in age, education, BMI and brain carotenoid, *α*-tocopherol, and retinol concentrations between the GDRS groups. Chi-square tests were used for categorical variables, which included sex, race, living arrangement, smoking status, alcohol use, hypertension, and diabetes. Repeated measures ANOVA was used to determine differences in carotenoids, tocopherol, and retinol concentrations between the four regions of the brain. For frontal and occipital cortices, tissue from both the left and right lobes of the brain was obtained. For cerebellum and temporal cortices, tissue from only the right side of the brain was obtained. No differences were observed in carotenoid, tocopherol and retinol concentrations between the right and left lobes for ten decedents (data not shown). In order to maintain consistency, only the right lobe of the brain was analyzed for all decedents. Mean brain carotenoid, *α*-tocopherol, and retinol concentrations were calculated for each decedent based on measures from the four regions of the brain (cerebellum frontal, occipital, and temporal cortices). These means were used for comparison of carotenoid, *α*-tocopherol, and retinol profiles between the brain and serum and also to evaluate differences between brain concentrations of individual micronutrients. Partial correlation coefficients were determined in order to evaluate the relationship of carotenoids, *α*-tocopherol, and retinol with different measures of cognitive function. Age, sex, education, diabetes, and hypertension were used as covariates since these variables have the strongest influence on cognitive function measures. Concentration of *trans* lutein and zeaxanthin in the cerebellum was significantly greater than the three cortical regions of the brain. In order to determine associations with cognitive indices, concentration of carotenoids in the temporal, frontal, and occipital cortices was averaged, and associations were evaluated with and without cerebellum carotenoids.

## 3. Results

### 3.1. Serum Analytes and Cognition

#### 3.1.1. Subject Characteristics

The characteristics of the octogenarians and centenarians who provided serum are given in Table 1. A significantly greater proportion of the centenarians were women, institutionalized, and nonsmokers compared to the octogenarians (*P* < 0.001). Furthermore, the centenarians had significantly less education years, alcohol use, and BMI (*P* < 0.001). There was no difference in the prevalence of hypertension in these two age groups.

A greater proportion of the institutionalized subjects were women and nonsmokers compared to the community dwelling subjects (*P* < 0.02 and 0.035, resp., Table 1). They were also significantly older than the community dwelling subjects (*P* < 0.001). The institutionalized subjects had significantly less education years, alcohol use, and BMI (*P* < 0.001). There was no difference in the prevalence of hypertension in these two groups.

#### 3.1.2. Serum Carotenoids, *α*-Tocopherol, and Retinol Concentrations

Serum concentrations of individual carotenoids, *α*-tocopherol and retinol are given in Table 2. Compared to the octogenarians, the centenarians had lower mean values for all carotenoids and *α*-tocopherol, which was significantly different (*P* < 0.05) for all analytes except *cis* zeaxanthin and marginally significant for *trans* lutein (*P* < 0.075) and *β*-carotene (*P* < 0.084). Similarly, the institutionalized subjects had lower mean values for all carotenoids and *α*-tocopherol which was significantly different (*P* < 0.05) for all analytes except for cryptoxanthin, *α*-carotene, and *β*-carotene and marginally significant for *trans* zeaxanthin (*P* < 0.052). Serum retinol values were neither significantly different between octogenarians and centenarians nor between community dwelling and institutionalized subjects.

#### 3.1.3. Cognitive Function in Octogenarians and Centenarians

The cognitive function status of subjects from the Georgia Centenarian Study is found in Table S2 of the Supplementary Material. For all measures of cognitive function, mean values were significantly lower in the centenarians than in octogenarians (*P* < 0.0001) except for delayed recognition which was not different between the two groups. For all measures of cognitive function, the institutionalized subjects had significantly lower values than the community dwelling subjects (*P* < 0.0005).

#### 3.1.4. Relationships between Serum Carotenoids, *α*-Tocopherol, and Retinol and Cognitive Performance

In the total study population, serum lutein and zeaxanthin concentrations were most consistently related to better cognitive performance, with a significant correlation observed (*P* < 0.05) (Table 3) for all cognitive measures except delayed recognition. It should be noted that none of the serum analytes were correlated with delayed recognition. Serum concentrations of *β*-carotene were also significantly correlated to most measures of cognitive function (*P* < 0.05) with the exception of the MMSE and delayed recognition (Table 3). Serum *α*-tocopherol concentrations were inversely related to dementia severity (Geriatric Deterioration Scale) (*P* < 0.01) and positively related to delayed retention, abstract reasoning, and the Behavioral Dyscontrol Scale (*P* < 0.05). Higher serum lycopene concentrations were only related to a lower dementia severity (*P* < 0.01). Serum cryptoxanthin was only related to the WAIS-III test (*P* < 0.05, Table 5). Serum retinol concentrations were not related to any of the cognitive function measures.

In the octogenarians, serum lutein concentrations were significantly related to measures of global cognition, lower dementia severity, and executive function (*P* < 0.05) (Table S3 of the Supplementary Material). In this age group, serum cryptoxanthin was inversely related to delayed recall (*P* < 0.05). There were no other significant relationships. In the centenarians, none of the serum carotenoids or *α*-tocopherol were related to global cognition or delayed recognition. Higher concentrations of lutein, zeaxanthin, *β*-carotene, and *α*-tocopherol were significantly related to lower dementia severity (*P* < 0.05). Additional significant relationships were found between lutein and abstract reasoning, between *β*-carotene and verbal fluency (Controlled Oral Word Association Test), WAIS-III and executive function, between *α*-tocopherol and executive function, and between retinol and delayed recall (*P* < 0.05).

In the community dwelling subjects, serum zeaxanthin had significant relationships with most measures of cognitive function (Supplementary Material, Table S4), with higher concentrations being significantly related to global cognitive performance, lower dementia severity, delayed recall and retention, verbal fluency, and concept formation/abstraction (*P* < 0.05). Higher serum lutein was significantly related to global cognitive function, lower dementia severity, and delayed recall and retention (*P* < 0.05). Other significant relationships were found between higher *β*-carotene concentrations and lower dementia, delayed recall and retention, and verbal fluency and executive function (*P* < 0.05). In the community dwelling subjects, *α*-tocopherol was only significantly related to executive function (*P* < 0.05). There were no significant relationships found between cryptoxanthin, *α*-carotene and retinol and cognitive measures.

Fewer significant relationships were found between serum analytes and cognitive measures in the institutionalized subjects. In this group, significant relationships were found between lower dementia severity and serum concentrations of lutein, zeaxanthin, *β*-carotene, and *α*-tocopherol. Serum lutein, zeaxanthin, cryptoxanthin, and *α*-carotene were significantly related to concept formation/abstraction (*P* < 0.05). Lutein, zeaxanthin, *β*-carotene, *α*-tocopherol and retinol were significantly related to executive function (*P* < 0.05).

### 3.2. Brain Analytes and Cognition

#### 3.2.1. Subject Characteristics

The characteristics of the cententarian population for which brain tissues were available are described in Table S5 of the Supplementary Material. Of the 47 decedents, five had normal cognitive function (GDRS = 1), seven had subjective mild memory loss (GDRS = 2), and eleven had MCI (GDRS = 3). There were 24 decedents who had different stages of dementia (GDRS 4 to 7). Subjects with dementia were slightly older than those with intact cognitive function. However, the differences were not significant. Eighty-nine percent of the decedents were females, and 89% were Caucasians. Race/ethnicity, sex, education, BMI, smoking status, and alcohol use did not differ by GDRS status. A greater proportion of the decedents were institutionalized; however, the differences between institutionalized and community dwelling subjects were not statistically significant. The prevalence of diabetes and hypertension did not differ between these two groups.

#### 3.2.2. Brain Carotenoid, *α*-Tocopherol, and Retinol Concentrations

The mean concentration of individual carotenoids, *α*-tocopherol, and retinol in the cerebellum, frontal, occipital, and temporal cortices from the 47 decedents is shown in Table 4. The range of values for carotenoids was 0–661 pmol/g. For *α*-tocopherol, the range was 22,979–137,576 pmol/g and for retinol 202–2,233 pmol/g. Mean lutein and zeaxanthin concentrations were significantly greater in the cerebellum compared to the frontal, occipital, and temporal cortices. Concentrations of cryptoxanthin and *β*-carotene the were highest in the occipital cortex and were significantly different from the frontal and temporal cortices. *α*-Carotene was not detected in these brain tissues. Contrary to lutein and zeaxanthin, concentrations of *α*-tocopherol and retinol were the lowest in the cerebellum and significantly different from all three cortical regions.

The proportion of* cis *to *trans* isomers was much lower in the brain than in the serum. The ratio of *cis* to *trans* lutein and *β*-carotene was ~0.25 in the serum while in the brain it was only ~0.04. Although present in the serum, *cis* isomers of zeaxanthin and lycopene were not detected in any of the brain regions analyzed. Of note is that brain carotenoids were significantly related to their concentrations in serum (*P* < 0.01 for all, except *cis* lutein: *P* < 0.05). *α*-Tocopherol concentrations in the cortices were also significantly related to serum concentrations (*P* < 0.01). However, *α*-tocopherol in the cerebellum was not. Retinol concentrations in all brain regions were not related to serum retinol concentrations.

**Figure 1:**
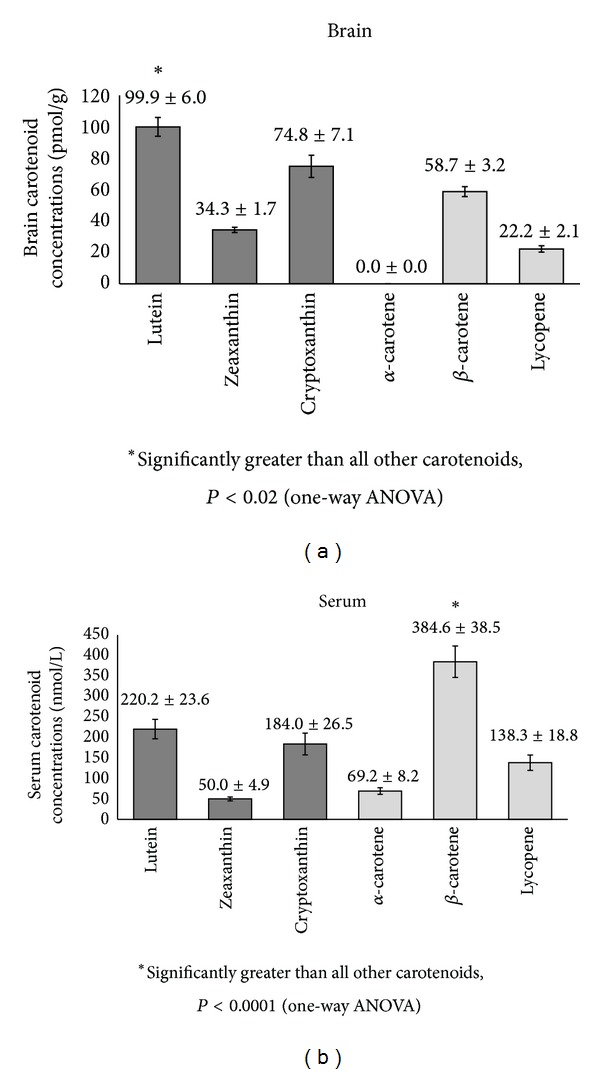
Mean (±SEM) concentrations of carotenoids (*trans* isomers) in the brain (average of cerebellum frontal serum, occipital, and temporal cortices) of decedents from the Georgia Centenarian Study (*n* = 42). Dark gray bars indicate xanthophyll carotenoids. Light gray bars indicate carotenes.

Figure 1 shows the mean carotenoid (*trans* isomers) concentrations in the brain and matched serum for the decedents that had both serum and brain tissue. In the brain, xanthophylls (lutein, zeaxanthin, and cryptoxanthin) accounted for 72% of total carotenoids, of which lutein accounted for 34% of the total and was significantly greater than all other carotenoids (*P* < 0.02). The proportion of carotenes (*α*-carotene, *β*-carotene, and lycopene) was higher than xanthophylls in serum, accounting for 57% of the total carotenoids of which *β*-carotene accounted for 37% of the total and was significantly greater than all other carotenoids (*P* < 0.0001).

#### 3.2.3. Brain Carotenoids, *α*-Tocopherol, and Retinol Concentrations in Decedents with Intact Cognitive Function, Mild Memory Loss, and MCI

The mean brain concentrations of carotenoids, *α*-tocopherol, and retinol for decedents with GDRS ≤ 3 and GDRS > 3 were not significantly different (data not shown) with the exception of 9 *cis β*-carotene which was significantly higher in dementia (17 ± 3.1 versus 9,8 ± 1.7 pmol/g).

Table 5 shows brain carotenoids, *α*-tocopherol, and retinol concentrations in decedents with normal cognitive function, mild memory loss, and MCI. Due to the advanced pathological changes in brain and significant reduction in brain volume associated with dementia, decedents with dementia were not included in this analysis [43]. Additionally, the neuropathological and neurobiological brain changes associated with MCI are quantitatively less than those associated with dementia [44]. Mean concentrations of all carotenoids were found to progressively decrease with increasing GDRS scores from 1 to 3. However, only the differences in lutein and *β*-carotene concentrations between subjects with normal cognitive function and MCI were statistically significant (*P* < 0.05). When data were adjusted for age, sex, education, diabetes, and hypertension, only the differences observed in lutein concentrations between the two groups remained significant (*P* < 0.05). Mean brain retinol concentration was not significantly different between individuals with MCI and normal cognitive function. Similar results were obtained when concentrations of carotenoids, *α*-tocopherol, and retinol in the cerebellum and cortex (average of frontal, temporal, and occipital) were analyzed separately. Data corrected for covariates are not reported due to the small sample size in each GDRS subgroup.

#### 3.2.4. Relationship between Brain Carotenoids, *α*-Tocopherol, and Retinol and Cognitive Function Measures

Of the 23 decedents with normal cognitive function, mild memory loss, and MCI, data for 21 subjects whose cognitive function tests were performed within one year (4.3 ± 2.8 mo, range: 0.3–10.5 mo) prior to their death were analyzed for associations with cognition. A significant positive correlation was observed between lutein concentrations in the cortex and the MMSE (global measure of cognitive function) and the Boston Naming Test (CERAD battery, a measure of language), while a negative correlation was observed with Geriatric Depression Scale (*P* < 0.05) (Table 6). The positive association of zeaxanthin in the cortex and verbal fluency was statistically significant (*P* < 0.05). *α*-Tocopherol was positively associated with SIB (a global measure of cognitive function) and COWAT (a measure of executive function) (*P* < 0.05). In the case of retinol, there was a negative relation with FOME-delayed recognition (*P* < 0.05). No associations were observed with the other listed measures of cognitive function.

Carotenoids in the cerebellum were not associated with any of the cognitive function measures with the exception of a negative association between lutein and the Geriatric Depression Scale (*r* = −0.63,  *P* = 0.005). *α*-tocopherol in the cerebellum was positively associated with MMSE (*r* = 0.535, *P* = 0.027) and SIB (*r* = 0.602, *P* = 0.01), both of which are measures of global cognition.

## 4. Discussion

In this cross-sectional study involving octogenarians and centenarians, we found significant relationships between serum and brain concentrations of dietary carotenoids and various measures of cognitive function. No specific domain of the cognitive performance showed the strongest relationship with either serum or brain concentrations of carotenoids since significant relationships were observed with memory, executive function, and language. The fact that specific carotenoids were associated with more than one cognitive function and that these associations remained statistically significant after controlling for potential confounding factors supports a possible role for these phytonutrients in age-related cognitive health. The present study is the first report on serum and brain carotenoids, *α*-tocopherol, and retinol concentrations and their relationship to cognitive function in the oldest of the old. This is of importance given the dramatic increase in the number of Americans surviving into their 80s and 90s and the increased prevalence of age-related cognitive diseases such as Alzheimer's disease [4].

Others have also found relationships with dietary carotenoids and age-related cognitive function. In cross-sectional and longitudinal analysis of 442 subjects aged 65–94 years, Perrig et al. [45] reported that a higher *β*-carotene plasma level was associated with better memory performances (free recall, recognition, and vocabulary). In the Rotterdam Study of 5182 community participants aged 55–95 yrs, cross-sectional analysis found that a lower intake of *β*-carotene was associated with impaired cognitive function as measured by the MMSE [46]. In both of these studies, there were no significant relationships with vitamin E. Also, no other carotenoids were evaluated. Two studies have reported that supplementation with antioxidants including *β*-carotene [47, 48] reduces the risk of cognitive decline. However, given that both studies involved multivitamin/mineral supplementation, a specific effect of *β*-carotene is difficult to determine. In the EVA, a cross-sectional study using a variety of cognitive measures in 589 subjects (68–79 yrs), it was found that those with the lowest cognitive functioning (<25th percentile) had a higher probability of having low plasma levels of lycopene and zeaxanthin, but not lutein or *β*-carotene [49]. However, in a prospective study of older adults (mean age 73 y), Morris and colleagues found no association between *β*-carotene intake and risk of Alzheimer's disease [50]. Additionally, the Age-Related Eye Diseases Study Research Group [51] found no effect of a multivitamin/mineral supplementation which included *β*-carotene on MMSE score and a battery of cognitive measures in a population with a median age of 69 yrs. Rinaldi et al. found that plasma levels of lutein, zeaxanthin, and *α*-carotene were lower in MCI and Alzheimer's disease subjects compared to controls but no difference for lycopene and *β*-carotene [10]. *β*-Cryptoxanthin was also significantly lower than controls in subjects with Alzheimer's disease but not in subjects with MCI. Others have reported lower levels of zeaxanthin, *β*-cryptoxanthin, lycopene, and *β*-carotene but not lutein and *α*-carotene in Alzheimer's patients than in controls [52]. Inconsistencies among studies may be due to limited sample size, cognitive tests used, method of carotenoid assessment, or characteristics of the subject population. The major difference between our present study and previous research is the age of the population. In our population, the average age was 97 yrs compared to averages of 67–77 yrs for the studies discussed above.

Whether a possible protective effect of carotenoids differs between the old and the oldest of the old remains to be tested. However, in our study, the most consistent relationships with cognition were observed for serum lutein, zeaxanthin, and *β*-carotene, reflecting diets rich in green leafy vegetables and orange and yellow vegetables such as carrots, sweet potatoes, and winter squash [53]. This remained to be true for the centenarians and with respect to living status, but only lutein remained significantly related to better measures of cognitive performance in the octogenarians. Furthermore, in brain tissue only concentrations of lutein and *β*-carotene were significantly lower in the cortex and cerebellum of subjects with MCI compared to those with normal cognitive function. Lutein and *β*-carotene may thus be important carotenoids for maintaining normal cognitive function in older adults. Consumption of vegetables, particularly the green leafy varieties that are rich sources of lutein and *β*-carotene, was associated with slower rates of cognitive decline in two large cohort studies [5, 8]. Other evidence suggests that lutein supplementation, alone or in combination with docosahexaenoic acid, may be able to improve certain aspects of cognitive performance in healthy older women [22].

Although much recent work has focused on lutein and its role in ocular health [54], lutein was also the dominant carotenoid in various regions of the centenarian brains. On the contrary, carotenes (*α*-carotene, *β*-carotene, and lycopene) were predominant in serum, which more closely reflects dietary intake. These findings suggest that although not predominant in the diet, there seems to be a preferential uptake of lutein from the circulation into the brain. Craft et al. reported similar preferential uptake of xanthophylls in brain [23]. *Trans* isomers of lutein, zeaxanthin, cryptoxanthin and *β*-carotene, *α*-tocopherol, and retinol were detected in all the brain tissues analyzed in this study. Only three *cis* isomers, two of lutein and one of *β*-carotene, were detected in the centenarian brain, which were not reported in the elderly brain tissues analyzed by Craft et al. [23]. Also, the ratios of *cis* to *trans* isomers in the brain were much lower than in serum, indicating a preferential uptake of *trans* isomers in the brain of these centenarians.

The majority of our findings find an association between lutein concentrations in serum or brain with age-related cognitive performance. However, there were significant associations for zeaxanthin and *β*-carotene as well. Therefore, the protective effect of carotenoids does not appear to be limited to the provitamin A carotenoids (*β*-cryptoxanthin, *α*-carotene, and *β*-carotene) nor to a class of carotenoids (xanthophylls versus carotenes). In addition, brain retinol levels were positively related to very few of the measures of cognition and negatively related to delayed recognition.

A protective effect of carotenoids may, in part, to be related to an antioxidant effect given that an antioxidant function is common to all carotenoids [55–58]. Furthermore, *α*-tocopherol, a major dietary antioxidant, was found to be related to several measures of cognitive. Cortical carotenoids may be protective in nature and may also influence interneuronal communication and function via multiple mechanisms. Other mechanisms by which certain carotenoids may function include modulation of functional properties of synaptic membranes along with changes in their physicochemical and structural features [59]. Carotenoids have also been shown to enhance gap junctional communication [60] which in the retina is important for light processing and may be important for the development of neural circuitry in the visual system. Lutein and zeaxanthin, as macular pigments in the retina, have been related to increased visual processing speed and to reduced scotopic noise (noise associated with vision under dim light conditions) [61–63]. Lutein may also have anti-inflammatory action in the brain, lowering inflammatory markers, and preventing cognitive decline [64, 65]. Neuroinflammation is also one of the factors that contribute to the pathogenesis of MCI and AD with increased levels of inflammatory markers being correlated with cognitive impairment [66].

The significant progressive decline in brain lutein and *β*-carotene with increased impairment from normal to MCI indicates that these carotenoids may play a role in preventing cognitive decline. MCI is thought to be the transitional stage between normal aging and the earliest symptoms of AD. There may be as much as a 50% likelihood of individuals with MCI developing AD within five years [67]. Our finding of a significant decrease in lutein and *β*-carotene in subjects with MCI indicates that these carotenoids may play a role in maintenance of cognitive health prior to a decline to MCI. Future clinical studies should focus on nutritional interventions with lutein and *β*-carotene in subjects with MCI. Thus, far supplementation studies with *β*-carotene have yielded mixed results; the Physician Health Study II showed a long-term beneficial effect [68], while some studies showed no effect on cognitive function with *β*-carotene or antioxidant supplementation [69, 70]. Effects of lutein supplementation on subjects with MCI have not been studied to date.

The strength of this study is that we were able to evaluate cognitive function in the elderly using a battery of cognitive tests that included the MMSE. This approach is more powerful than using only the less sensitive measure of MMSE. With the other tests, the scores ranges are wider, allowing for a better ability to study cognitive impairment. We were also able to evaluate cognitive relationships with a variety of dietary carotenoids using measures of serum concentrations. Carotenoid assessment using serum concentrations may be preferred to food frequency questionnaires because the high interindividual variation in intestinal bioavailability of carotenoids [71] does not need to be considered. Whether such variability exists for, uptake of carotenoids into brain tissue is not known. What is known is that the macular pigment response to supplementation of lutein from diet or supplements varies widely among individuals [72, 73]. A variable response of uptake into the brain may also exist given the significant relationship between concentrations of xanthophylls in the retina and brain [26].

Thus, another strength of this study is the measures in brain tissue that are associated with premortem measures of cognition. Additionally, the strength of these cross-sectional relationships lies in the high interindividual variability in brain carotenoid concentrations, similar to that observed in the serum of these centenarians. Another strength of the study is that associations with cognitive function were evaluated with carotenoids, *α*-tocopherol, and retinol actually embedded in brain tissue. However, the significant correlations between carotenoid concentrations in serum and brain tissue suggest that serum carotenoid measures could be a useful tool for evaluating the benefits of dietary carotenoids to age-related cognitive health.

One limitation to this study is that, in cross-sectional analysis, it is not possible to affirm whether these low levels of carotenoids preceded or were the consequence of cognitive impairment. Low carotenoid status, that is, poor food choices, may be a reflection of poor cognitive status. The significant relationships observed may not be due to specific effects of the individual carotenoids but, indication of overall healthier diets and lifestyles. Another limitation is that our analyses were performed in a sample drawn from a relatively small population. Therefore, we were unable to perform multivariate regression analysis that would have provided additional information regarding the independent contributions of the individual nutrient components.

## 5. Conclusions

In conclusion, this is the first study to evaluate the role of carotenoids, *α*-tocopherol, and retinol in cognitive function in the oldest of the old. To date, previous studies evaluated older populations with average ages of 20–30 years younger than those of this study. Evaluations of the role of diet to health in this age group are becoming increasingly important given the rise in both life expectancy and the segment of the population who are >80 years. While far from conclusive, the idea that certain carotenoids can influence cognitive function is certainly feasible. The significance of our findings requires further research using biological studies, longitudinal epidemiological studies, and clinical trials with carotenoid supplementation.

## Supplementary Material

The Supplemental Material provides details on the cognitive measures as well as their relationships with serum concentrations of carotenoids in the Georgia Centenarian Study population. Characteristics of the centenarian subjects in the analysis of brain analytes and cognition is also provided.

## Figures and Tables

**Table 1:** Subject characteristics.

	≥80 to ≤89 y (*n* = 78)	≥98 y (*n* = 220)	*P* value*	Community dwelling (*n* = 150)	Institutionalized (*n* = 148)	*P* value*	Total (*n* = 298)
Age, yrs (mean ± SD)	84.2 ± 2.7	100.4 ± 1.9		93.3 ± 8.3	99.7 ± 4.6	<0.0001	96.6 ± 7.4
Male : female	27 : 51	36 : 184	0.0007	40 : 110	23 : 125	0.0187	63 : 235
Community dwelling (*n*) : institutionalized (*n*)	66 : 12	84 : 136	<0.0001	—	—		150 : 148
Education, yrs (mean ± SD)	13.0 ± 3.5	10.6 ± 3.8	<0.0001	12.1 ± 3.7	10.3 ± 3.7	<0.0001	11.2 ± 3.8
Body mass index (kg/m^2^) (mean ± SD)	25.5 ± 4.8	22.6 ± 4.7	<0.0001	24.2 ± 4.7	22.5 ± 4.8	0.0059	23.3 ± 4.8
Smoking (*n*)			<0.0001			0.035	
None	33	156		88	101		189
Ex-smoker	40	54		53	41		94
Current	5	7		9	3		12
No information	0	3		0	3		3
Alcohol use (*n*)			<0.0001			<0.0001	
None	22	134		67	89		156
Ex-drinker	27	45		32	40		72
Current	29	39		51	17		68
No information	0	2		0	2		2
Hypertension (*n*)			0.394			0.306	
Yes	32	108		66	74		140
No	45	109		83	71		154
No information	1	3		1	3		4

*Significant difference between groups. Means were compared using Student's *t*-test. Fisher's exact test was applied for categorical variables.

**Table 2:** Serum carotenoid, *α*-tocopherol, and retinol concentrations in subjects from the Georgian Centenarian Study (mean ± SD).

nmol/L (carotenoids) *μ*mol/L (*α*-tocopherol, retinol)	≥80 to ≤89 y (*n* = 78)	≥98 y (*n* = 220)	*P* value*	Community dwelling (*n* = 150)	Institutionalized (*n* = 148)	*P* value*	Total (*n* = 298)
Lutein, *trans *	213 ± 162	199 ± 177	0.0753	223 ± 199	181 ± 141	0.0072	204 ± 174
Lutein, *cis *	58 ± 58	53 ± 65	0.0006	63 ± 77	46 ± 46	0.0162	56 ± 63
Lutein, total	316 ± 255	293 ± 285	0.0156	334 ± 329	265 ± 207	0.0116	300 ± 276
Zeaxanthin, *trans *	47 ± 23	44 ± 28	0.0194	49 ± 30	42 ± 23	0.0516	46 ± 26
Zeaxanthin, *cis *	5 ± 16	5 ± 11	0.6388	7 ± 14	4 ± 9	0.0764	5 ± 12
Zeaxanthin, total	53 ± 32	49 ± 37	0.0355	56 ± 40	46 ± 28	0.0195	51 ± 35
Cryptoxanthin	159 ± 105	148 ± 125	0.0362	166 ± 137	134 ± 96	0.3174	150 ± 119
*α*-Carotene	80 ± 110	63 ± 61	0.0122	71 ± 93	63 ± 54	0.6972	67 ± 76
*β*-Carotene, *trans *	547 ± 836	443 ± 419	0.2200	542 ± 670	398 ± 406	0.0056	471 ± 558
*β*-Carotene, *cis *	20 ± 26	17 ± 22	0.6538	19 ± 26	17 ± 20	0.5322	19 ± 22
*β*-Carotene, total	568 ± 855	460 ± 432	0.0839	560 ± 69	415 ± 419	0.0039	488 ± 573
Lycopene, *trans *	240 ± 181	138 ± 119	<0.0001	199 ± 166	130 ± 112	<0.0001	164 ± 145
Lycopene, *cis *	389 ± 277	231 ± 227	<0.0001	324 ± 276	220 ± 212	0.0003	272 ± 251
Lycopene, total	629 ± 443	369 ± 337	<0.0001	523 ± 430	348 ± 309	0.0003	436 ± 384
*α*-Tocopherol	31.9 ± 16.8	25.5 ± 12.9	0.0011	29.6 ± 15.0	24.8 ± 13.2	0.0016	29.2 ± 14.3
Retinol	2.14 ± 0.07	1.84 ± 0.62	0.449	2.00 ± 0.64	1.83 ± 0.59	0.247	1.92 ± 0.62

*Significant difference between groups. Means were compared using Student's *t*-test. Chi-square analysis was applied for categorical variables.

**Table 3:** Partial correlation coefficients between cognition indices and serum carotenoids, *α*-tocopherol, and retinol in octogenarians and centenarians from the Georgia Centenarian Study (adjusted w/age, sex, education years, BMI, smoking, alcohol, hypertension, and diabetes, *n* = 298).

	Mini-Mental State Exam	Global Deterioration Rating Scale	FOME^1^ delayed recall	FOME delayed recognition	FOME delayed retention	Controlled Oral Word Association Test	WAIS-III Similarities Subtest^2^	Behavioral Dyscontrol Scale Total Score
Lutein, (*trans* + *cis*)	0.114	−0.350^a^	0.177^b^	0.009	0.114	0.148^c^	0.244^a^	0.180^b^
Zeaxanthin, (*trans* + *cis*)	0.128^c^	−0.236^a^	0.134^c^	0.069	0.146^c^	0.169^b^	0.184^b^	0.196^b^
Cryptoxanthin	0.026	−0.096	0.054	0.002	0.035	0.031	0.129^c^	0.045
*α*-Carotene	−0.014	−0.068	0.017	−0.029	0.009	0.015	0.074	0.029
*β*-Carotene, (*trans* + *cis*)	0.072	−0.244^a^	0.173^b^	0.082	0.168^b^	0.186^b^	0.144^c^	0.227^a^
Lycopene, (*trans* + *cis*)	−0.014	−0.142^c^	−0.017	−0.033	0.014	0.017	0.108	0.044
*α*-Tocopherol	0.118	−0.194^b^	0.097	0.097	0.145^c^	0.106	0.121^c^	0.200^b^
Retinol	0.089	−0.078	0.113	0.022	0.105	0.006	0.045	0.116

^1^FOME: Fuld Object Memory Evaluation; ^2^WAIS-III Similarities Subtest: Wechsler Adult Intelligence Scale-III Similarities Subtest.

Significantly related (*P*<): ^a^0.001; ^b^0.01, ^c^0.05.

**Table 4:** Mean (±SEM) concentrations of carotenoids, *α*-tocopherol, and retinol in the cerebellum, frontal, occipital, and temporal cortices (*n* = 47).

Analyte (pmol/g)	Cerebellum	Frontal cortex	Occipital cortex	Temporal cortex
Lutein, *cis *	6.5 ± 1.4^a^	4.0 ± 1.6^a,b^	2.8 ± 0.7^b^	3.1 ± 0.8^b^
Lutein, *trans *	169.8 ± 15.5^a^	78.7 ± 7.6^b^	91.8 ± 9.1^b^	81.7 ± 7.8^b^
Total Lutein (*cis *+ *trans*)	176.4 ± 16.6^a^	82.7 ± 8.0^b^	94.6 ± 9.6^c^	84.8 ± 8.4^b,c^
Zeaxanthin, *trans *	52.9 ± 4.3^a^	25.9 ± 1.9^b^	30.0 ± 2.0^c^	27.8 ± 2.0^b,c^
Cryptoxanthin (*α* + *β*)	75.8 ± 13.7^a,b^	63.4 ± 10.2^a^	93.4 ± 15.6^b^	60.7 ± 9.4^a^
*β*-Carotene, *trans *	59.8 ± 5.7^a^	60.8 ± 6.4^a^	70.2 ± 6.9^b^	51.8 ± 4.3^a^
*β*-Carotene, 9-*cis *	12.0 ± 1.5^a^	13.5 ± 1.8^a^	16.9 ± 2.0^b^	13.1 ± 1.9^a,b^
Lycopene, *trans *	26.3 ± 4.4	21.0 ± 3.7	21.9 ± 3.6	19.9 ± 3.2
*α*-Tocopherol	43475 ± 1877^a^	67027 ± 2992^b,c^	72971 ± 2442^c^	65521 ± 1940^b^
Retinol	472 ± 24^a^	615 ± 42^b^	676 ± 43^b,c^	768 ± 54^c^

Means not sharing a common superscript in the same row are significantly different at *P* < 0.05 (repeated measures ANOVA with Bonferroni adjustment for multiple comparisons).

**Table 5:** Mean (±SEM) concentrations of carotenoids, *α*-tocopherol, and retinol in the brain (average of cerebellum, frontal, occipital, and temporal cortices) based on premortem GDRS scores in decedents with normal cognitive function (GDRS = 1), subjective mild memory loss (GDRS = 2), and mild cognitive impairment (GDRS = 3).

pmol/g	Global deterioration scale (GDRS)^a^		
	1 (*n* = 5)	2 (*n* = 7)	3 (*n* = 11)
Lutein, *trans *	133 ± 21^b^	124 ± 17^b,c^	67 ± 14^c^
Total lutein (*cis* + *trans*)	145 ± 22^b^	127 ± 18^b,c^	68 ± 15^c^
Zeaxanthin, t*rans *	45.0 ± 7.5	43.1 ± 6.3	25.9 ± 5.0
Cryptoxanthin	90.1 ± 22.4	63.2 ± 19.0	57.2 ± 15.1
*β*-Carotene, *trans *	77.6 ± 10.5^b^	48.0 ± 8.9^b,c^	39.5 ± 7.1^c^
Lycopene, *trans *	37.0 ± 9.0	26.1 ± 7.6	16.4 ± 6.1
*γ*-Tocopherol	1609 ± 370	2129 ± 313	1518 ± 249
*α*-Tocopherol	67408 ± 5295	63205 ± 4475	60028 ± 3570
Retinol	572 ± 109	530 ± 92	691 ± 74

^a^GDRS = 1: no subjective complaints or objective evidence of memory deficits; GDRS = 2: subject complaints, but no objective evidence of memory deficits; GDRS = 3: mild cognitive impairment.

Means not sharing a common superscript in the same row are significantly different at *P* < 0.05 (univariate ANOVA with Bonferroni adjustment for multiple comparisons).

**Table 6:** Cross-sectional relationship between concentrations of carotenoids, *α*-tocopherol, and retinol in the cortex (average concentrations of the frontal, occipital, and temporal cortices) and premortem measures of cognitive function in subjects with normal cognitive function, mild memory loss, and MCI who completed the cognitive function tests a year prior their death (*n* = 21).

	MMSE	FOME recognition	SIB	COWAT	BDS	CERAD verbal fluency	CERAD Boston naming test
Lutein (*trans* + *cis*)	0.494^a^	−0.297	0.076	0.142	0.459	0.455	0.572^a^
Zeaxanthin	0.439	−0.281	0.139	0.177	0.286	0.461^a^	0.207
Cryptoxanthin	−0.056	0.325	−0.021	0.152	0.046	0.302	0.084
*β*-Carotene, *trans *	0.265	−0.010	−0.014	0.269	0.137	0.265	0.008
Lycopene, *trans *	0.124	−0.003	0.132	0.067	−0.085	0.065	0.275
*α*-Tocopherol	0.393	−0.295	0.568^a^	0.637^a^	0.434	0.328	0.165
Retinol	0.161	−0.456	0.168	0.400	0.067	0.108	−0.307

Values are partial correlation coefficients adjusted for sex, education, diabetes, and hypertension.

^a^ *P* ≤ 0.05.

MMSE: Mini-Mental State Examination; FOME: Fuld Object Memory Evaluation; SIB: Severe Impairment Battery; COWAT: Controlled Oral Word Association Test; BDS: Behavioral Dyscontrol Scale; CERAD: Consortium to Establish a Registry for Alzheimer's Disease.

## Abbreviations

BMI: Body mass index
BDS: Behavioral Dyscontrol Scale
CERAD: Consortium to Establish a Registry for Alzheimer's Disease
COWAT: Controlled Oral Word Association Test
FOME: Fuld Object Memory Evaluation
GCS: Georgia Centenarian Study
GDRS: Global deterioration rating scale
HPLC: High-performance liquid chromatography
MTBE: Methyl tert-butyl ether
MCI: Mild cognitive impairment
MMSE: Mini-Mental State Examination
MP: Macular pigment
SIB: Severe Impairment Battery
WAIS-III: Wechsler Adult Intelligence Scale-III.
