# Supplementary material for: Relationship between Serum and Brain Carotenoids, α-Tocopherol, and Retinol Concentrations and Cognitive Performance in the Oldest Old from the Georgia Centenarian Study
Source: J Aging Res. 2013 Jun 9;2013:951786. doi: 10.1155/2013/951786 (PMC3690640; doi:10.1155/2013/951786)
Supplement: Supplementary file 1 — The Supplemental Material provides details on the cognitive measures as well as their relationships with serum concentrations of carotenoids in the Georgia Centenarian Study population. Characteristics of the centenarian subjects in the analysis of brain analytes and cognition is also provided. [file 951786.f1.pdf]

Table S1. Cognitive tests used in the Georgia Centenarian Study.

Test	Assessment	Description	Ref.
Mini-Mental State Examination	Global cognitive performance	A brief screening of orientation, memory, executive function, language, praxis	(1)
Global Deterioration Rating Scale	Dementia severity	Rating scale of dementia stages	(2)
Severe Impairment Battery	Global cognitive performance (significantly cognitively impaired individuals)	Direct performance-based assessment of a variety of low-level tasks	(3)
Fuld Object Memory Evaluation			
delayed recall	delayed recall	Number of objects recalled after a 20 min delay	(4)
delayed recognition	delayed recognition	Objects not recalled that were recognized	
delayed retention	delayed retention	Sum of delayed recall and recognition	
Controlled Oral Word Association Test	Verbal fluency	Participants are required to name as many words as they can in one minute	Adapted from: (5)
WAIS-III Similarities	Concept formation, abstraction	Participants are given a series of comparisons and are asked how two things are alike	(6)
Behavioral Dyscontrol Scale	Executive function	A brief multidimensional screening of working memory, motor learning, and behavioral inhibition	(7)
Geriatric Depression Scale - Short Form	Depressive symptoms	Participants are asked a series of questions about how they felt over the past week.	(8)
Direct Assessment of Functional Status	Cognitive and behavioral functional status	Behavioral rating scale that directly assesses functional capacity	(9)
CERAD (Consortium to Establish a Registry for Alzheimer's Disease):	Clinical evidence for dementia	Standardized procedure for the clinical evaluation and diagnosis of patients with Alzheimer's disease (AD).	(10)

Verbal Fluency
Boston Naming Test
Mini-Mental State Examination
Constructional Praxis
Word List Memory

Table S2. Cognitive function status in subjects from the Georgia Centenarian Study (mean ± SD).

	≥80 to ≤89y (n=78)	≥98 y (n=220)	*P* value*	Community dwelling (n=150)	Institutionalized (n=148)	*P* value*	Total (n=298)
Mini Mental State Examination	24.6 ± 7.8	16.5 ± 8.7	<.0001	23.1±6.8	13.8±9.0	<.0001	18.4±9.3
Global Deterioration Rating Scale	2.4 ± 1.4	4.0 ± 1.7	<.0001	3.0±1.5	4.5±1.7	<.0001	3.7±1.8
FOME‡ Delayed Recall	6.4±3.1	3.0±3.1	<.0001	5.5±3.2	2.3±2.8	<.0001	3.9±3.4
FOME Delayed Recognition	2.4 ±2.0	3.1 ± 2.4	0.6547	3.2±2.1	2.7±2.6	0.0004	3.0±2.4
FOME Delayed Retention	8.7 ± 3.2	6.1 ± 4.0	0.0003	8.6±2.8	4.9±4.2	<.0001	6.7±4.0
Controlled Oral Word Association Test	7.9 ± 4.8	4.1 ± 3.6	<.0001	6.9±4.4	3.3±3.4	<.0001	5.1±4.3
Wechsler Adult Intelligence Scale-III Similarities subtest	15.2 ± 8.9	7.2 ± 8.1	<.0001	13.3±8.8	5.2±7.2	<.0001	9.2±9.0
Behavioral Dyscontrol Scale Total Score	14.9 ± 5.7	8.5 ± 6.4	<.0001	13.0±6.1	7.0±6.5	<.0001	1.0±7.0

‡FOME: Fuld Object Memory Evaluation; *Significant difference between groups. Means were compared using Student’s t-test.

Table S3. Partial correlation coefficients between cognition indices and serum carotenoids, α-tocopherol and retinol in octogenarians and centenarians (adjusted with age, sex, education years, BMI, smoking, alcohol, hypertension and diabetes).

	Mini Mental State Exam	Global Deterioration Rating Scale	FOME[1] Delayed Recall	FOME Delayed Recognition	FOME Delayed Retention	Controlled Oral Word Association Test	WAIS-III Similarities Subtest[2]	Behavioral Dyscontrol Scale Total Score
OCTOGENARIANS, n = 78								
Lutein, (*trans*+*cis*)	0.227[a]	-0.258[a]	0.172	-0.009	0.137	0.042	0.084	0.196[a]
Zeaxanthin, (*trans*+*cis*)	0.135	-0.059	-0.026	-0.079	-0.021	-0.087	0.069	0.078
Cryptoxanthin	-0.156	0.174	-0.189	-0.026	-0.105	-0.17	-0.063	-0.156
α-Carotene	-0.014	0.105	-0.016	-0.022	0.013	-0.059	-0.033	-0.014
β-Carotene, (*trans*+*cis*)	0.024	0.007	0.035	0.054	0.073	0.029	0.031	0.032
Lycopene, (*trans*+*cis*)	0.056	0.019	-0.063	-0.091	-0.069	-0.034	-0.024	0.005
α-Tocopherol	-0.065	0.022	-0.026	-0.002	-0.027	0.004	0.108	-0.065
Retinol	0.058	0.033	-0.006	0.072	0.039	-0.123	-0.031	0.091
CENTENARIANS, n = 220								
Lutein, (*trans*+*cis*)	0.066	-0.364[b]	0.164[a]	-0.007	0.094	0.157[a]	0.266[b]	0.153[a]
Zeaxanthin, (*trans*+*cis*)	0.085	-0.233[c]	0.139	0.084	0.157[a]	0.188[a]	0.182[a]	0.181[a]
Cryptoxanthin	0.047	-0.130	0.104	0.026	0.078	0.056	0.152[a]	0.064
α-Carotene	0.009	-0.133	0.045	-0.003	0.038	0.044	0.107	0.062
β-Carotene, (*trans*+*cis*)	0.042	-0.285[b]	0.203[b]	0.091	0.190[c]	0.211[c]	0.157[a]	0.240[c]
Lycopene, (*trans*+*cis*)	-0.074	-0.133	-0.031	-0.030	0.005	-0.009	0.108	0.015

α-Tocopherol	0.108	-0.213[c]	0.107	0.088	0.162[a]	0.114	0.09	0.228[c]
Retinol	0.100	-0.109	0.150[a]	0.012	0.124	0.068	0.081	0.114

[1]FOME: Fuld Object Memory Evaluation; [2]WAIS-III Similarities subtest: Wechsler Adult Intelligence Scale-III Similarities subtest

Significantly related (*P* <): [a]0.05, [b]0.001, [c]0.01

Table S4. Partial correlation coefficients between cognition indices and serum carotenoids, α-tocopherol and retinol in community dwelling (n = 150) and institutionalized subjects (n = 148), 80 -89 and ≥98 yrs (adjusted w/ age, sex, education years, BMI, smoking, alcohol, hypertension and diabetes).

	Mini Mental State Exam	Global Deterioration Rating Scale	FOME[1] Delayed Recall	FOME Delayed Recognition	FOME Delayed Retention	Controlled Oral Word Association Test	WAIS-III Similarities subtest[2]	Behavioral Dyscontrol Scale Total Score
COMMUNITY DWELLING, n = 150								
Lutein, (*trans*+*cis*)	0.220[a]	-0.330[b]	0.198[a]	-0.001	0.190[a]	0.164	0.112	0.113
Zeaxanthin, (*trans*+*cis*)	0.389[b]	-0.249[c]	0.172[a]	0.039	0.214[a]	0.191[a]	0.172[a]	0.164
Cryptoxanthin	0.01	-0.165	0.005	-0.091	-0.04	-0.041	0.086	0.04
α-Carotene	-0.016	-0.075	0.023	-0.075	0.042	-0.005	-0.029	0.021
β-Carotene, (*trans*+*cis*)	0.139	-0.224[c]	0.193[a]	0.136	0.313[b]	0.262[c]	0.094	0.286[b]
Lycopene, (*trans*+*cis*)	0.240[d]	-0.195[a]	0.032	-0.006	0.136	0.126	0.089	0.121
α-Tocopherol	0.147	-0.179[a]	0.076	0.071	0.145	0.133	0.155	0.189[a]
Retinol	0.094	-0.060	0.091	0.048	0.128	-0.048	0.029	0.025
INSTITUTIONALIZED, n = 148								
Lutein, (*trans*+*cis*)	0.047	-0.373[b]	0.132	-0.045	0.039	0.100	0.321[b]	0.184[a]
Zeaxanthin, (*trans*+*cis*)	0.034	-0.250[c]	0.093	0.044	0.097	0.153	0.216[a]	0.213[a]
Cryptoxanthin	0.025	-0.107	0.128	0.083	0.103	0.1	0.194[a]	0.074
α-Carotene	0.007	-0.107	0.064	0.049	0.041	0.056	0.189[a]	0.068

β-Carotene, (*trans*+*cis*)	0.010	-0.278[c]	0.154	0.004	0.075	0.131	0.151	0.190[a]
Lycopene, (*trans*+*cis*)	-0.095	-0.140	-0.066	-0.061	-0.037	-0.023	0.128	0.029
α-Tocopherol	0.086	-0.198[a]	0.065	0.044	0.106	0.062	0.064	0.190[a]
Retinol	0.075	-0.059	0.134	-0.010	0.087	0.030	0.070	0.177[a]

‡FOME: Fuld Object Memory Evaluation; [2]WAIS-III Similarities subtest: Wechsler Adult Intelligence Scale-III Similarities subtest

Significantly related (*P* <): [a]0.05, [b]0.001, [c]0.01

Table S5. Characteristics of centenarian subjects in the analysis of brain analytes and cognition.

Characteristics	Total	Global Deterioration Scale (GDRS)[a]				P value
		1	2	3	>3	
	n=47	n=5	n=7	n=11	n=24	
Age, y (mean ± SD)	101 ± 2	99 ± 1	100 ± 1	101 ± 2	101 ± 2	0.084
Sex,						
Male	5	0	3	1	1	0.104
Female	42	5	4	10	23	
Race,						
Caucasian	42	5	7	10	20	0.901
Black	5	0	0	1	4	
Living,						
Community dwelling	14	2	4	3	5	0.438
Institutionalized	33	3	3	8	19	
Education (mean ± SD) (highest grade completed, y)	10.3 ± 4.0	11.6 ± 3.2	11.7 ± 4.0	10.6 ± 4.7	9.5 ± 3.8	0.515
BMI, kg/m^2 (mean ± SD)	22.7 ± 5.8	24.4 ± 5.6	22.8 ± 2.7	22.8 ± 3.1	22.3 ± 7.4	0.918
Smoking (n)						
Never	30	5	4	9	12	0.732
Past	4	0	2	1	1	
Present	1	0	0	0	1	
Alcohol use (n)						
Never	21	2	2	5	12	0.247
Past	6	2	2	1	1	
Present	8	1	2	4	1	
Hypertension (n)						
Yes	25	4	4	4	13	0.640
No	22	1	3	7	11	
Diabetes (n)						
Yes	3	1	1	0	1	0.363
No	44	4	6	11	23	

[a]GDRS=1: no subjective complaints or objective evidence of memory deficits; GDRS=2: subject complaints, but no objective evidence of memory deficits; GDRS=3: mild cognitive impairment and GDRS=4 to 7: different stages of dementia.

Subject characteristics were compared using one-way ANOVA, for categorical variables Fisher's exact test was used.

REFERENCES (Supplementary Material)

1. Folstein MR, Folstein SE. "Mini-Mental State" A Practical Method for Grading the Cognitive State of Patients for the Clinician. Journal of Psychiatriatric Research 1975;12.
2. Reisberg B, Ferris SH, de Leon MJ, Crook. T. The global deterioration scale for assessment of primary degenerative dementia. American Journal of Psychiatry 1982;139:1136-1139.
3. Panisset M, Roudier M, Saxton J, Boller F. Severe impairment battery. A neuropsychological test for severely demented patients. Archives of Neurology 1994;51:41-5.
4. Fuld PA. Fuld Object Memory Evaluation instruction manual. Wood Dale, IL: Stoetling, 1981.
5. Benton AL, Hamsher K. Multilingual aphasia examination. Iowa City, Iowa: University of Iowa, 1976.
6. Wechsler D. Wechsler Adult Intelligence Scale-III. San Antonio, Texas: The Psychological Corporation, 1997.
7. Grigsby J, Kaye K, Robbins LJ. Reliabilities, norms and factor structure of the Behavioral Dyscontrol Scale. Perceptual & Motor Skills 1992;74:883-92.
8. Sheikh JI, Yesavage JA. Geriatric Depresion Scale:Recent evidence and development of a shorter version. Clinical Gerontology: A Guide to Assessment and Intervention. New York: The Haworth Press, 1986:165-173.
9. Loewenstein DA, Amigo E, Duara R, et al. A new scale for the assessment of functional status in Alzheimer's disease and related disorders. Journal of Gerontology 1989;44:P114-21.
10. Morris JC, Heyman A, Mohs RC, et al. The consortium to establish a registry for Alzheimer's disease (CERAD). Part I. Clinical and neuropsychological assessment of Alzheimer's disease. Neurology 1989;39:1159-1165.
